# Spatial and temporal variations of Rb/Sr ratios of the bulk surface sediments in Lake Qinghai

**DOI:** 10.1186/1467-4866-11-3

**Published:** 2010-07-09

**Authors:** Hai Xu, Bin Liu, Feng Wu

**Affiliations:** 1 State Key Laboratory of Loess and Quaternary Geology, Institute of Earth Environment, Chinese Academy of Sciences. Xi'an, 710075, China; 2 Graduate school of Chinese Academy of Sciences, Beijing 100049, China

## Abstract

The Rb/Sr ratios of lake sediments have been suggested as indicators of weathering intensity by increasing work. However, the geochemistry of Rb/Sr ratios of lake sediments is variable between different lakes. In this study, we investigated the spatial and temporal patterns of Rb/Sr ratios, as well as those of other major elements in surface sediments of Lake Qinghai. We find that the spatial pattern of Rb/Sr ratios of the bulk sediments correlates well with that of the mass accumulation rate, and those of the terrigenous fractions, e.g., SiO_2_, Ti, and Fe. The temporal variations of Rb/Sr ratios also synchronize with those of SiO_2_, Ti, and Fe of each individual core. These suggest that Rb/Sr ratios of the surface sediments are closely related to terrigenous input from the catchment. Two out of eight cores show similar trends between Rb/Sr ratios and precipitation indices on decadal scales; however, the other cores do not show such relationship. The result of this study suggests that physical weathering and chemical weathering in Lake Qinghai catchment have opposite influence on Rb/Sr ratios of the bulk sediments, and they compete in dominating the Rb/Sr ratios of lake sediments on different spatial and temporal scales. Therefore, it is necessary to study the geochemistry of Rb/Sr ratio of lake sediments (especially that on short term timescales) particularly before it is used as an indicator of weathering intensity of the catchment.

## Introduction

Rb generally coexists with K in the K-rich minerals, such as K-feldspar, and biotite, etc.; while Sr tends to enrich in Ca-bearing minerals, e.g. the limestone and the Ca-bearing silicates such as hornblende, plagioclase and picrite [1]. Since the Ca-bearing minerals are easy to breakdown compared with the K-bearing minerals, the K-Rb pair and Ca-Sr pair are easy to fractionate during natural processes [1,2]. For example the chemical weathering can leach the Ca-Sr much easier than K-Rb, which leads the residue enriched in K-Rb but depleted in Ca-Sr [1,2].

The different behaviors between Rb and Sr are useful in identifying material provenance and indicating the intensity of chemical weathering [1,3]. One example is the use of Rb/Sr ratio in the Chinese loess/paleosol profile as an indicator of intensity of Asian summer monsoon [3]. Since Sr-Ca can be leached much easier than Rb-K, the relict would have higher Rb/Sr ratio compared with the leached fraction. Therefore, as shown by Chen et al. [3,4], higher Rb/Sr ratios in a loess/paleosol profile correlated to higher degree of weathering, and thus to stronger monsoon precipitation on long-term timescales. On the other hand, the dissolved material is relatively rich in Sr but depleted in Rb compared with the parent rocks. These dissolved materials will be eventually transported into lakes/oceans and will therefore lead to lower Rb/Sr ratios in lake/ocean sediments. As a result, sediments with higher fraction of chemical/biogenic deposits have lower Rb/Sr ratios, while those with higher fraction of terrigenous detritus have higher Rb/Sr ratios. Some studies thus use the Rb/Sr ratios of lake sediments as a potential indicator of chemical weathering intensity in the catchment (e.g. [5]).

However, factors that influence the material load to lakes are quite complex, such as the local climates, the chemical/physical property of the bedrock, vegetation cover, and human activities, etc. These variable factors may complicate the environmental significance of the Rb/Sr ratios of lake sediments to a high extent. For example, the environmental changes can cause the Rb-Sr fractionation much easier for fresh rocks or slightly weathered rocks than highly-weathered rocks [1]. As a result, the chemical weathering may have stronger influence on the Rb/Sr ratios of lake sediments in a slightly weathered catchment; while the physical weathering may play a dominating role in a highly weathered catchment. For another scenario, if the physical loads in the catchment are very strong, even though the accompanied chemical weathering is expected to be enhanced, chemical composition of the lake sediments may be dominated by the terrigenous detritus but not the chemical/biogenic depositions. Under this circumstance, the Rb/Sr ratios of the bulk lake sediments should server as an indictor of physical weathering intensity, but not or only partly of chemical weathering intensity. Therefore, it is necessary to consider particularly the local processes, and investigate systematically the spatial and temporal distributions of the Rb/Sr ratio in lake sediments to make clear its environmental significance.

Lake Qinghai locates at the arid/semi-arid zone, NE Qinghai-Tibet plateau, northwest China. Understand of the material transportation from catchment into the lake is critical to evaluate and predict the ecology and environment, to make clear the environmental significance of some limnological indices, and to shed light on the weathering processes. Rb/Sr ratios in the lake sediments may provide such important information. However, to date the spatial and temporal variations of Rb/Sr ratios of sediments of Lake Qinghai is still unclear, and the environmental significance of Rb/Sr ratios needs to be evaluated. In this study we studied the spatial and temporal patterns of Rb/Sr ratios, as well as those of other major elements of the bulk surface sediments in Lake Qinghai in order to constrain the geochemistry of Rb/Sr ratios in sediments of Lake Qinghai.

## Background and method

### Bedrocks, climates, and hydrogeochemistry

**Figure 1:**
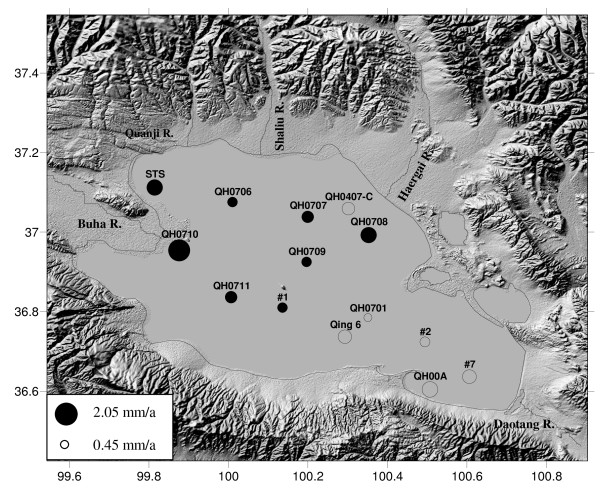
**Locations of the surface cores**. The black filled ones show the cores for which element contents were measured in this study. Sizes of the circles indicate the sedimentation rates derived from ^137^Cs radioactivities (ranged between 0.45~2.05 mm/a; see Table 1 in reference [7]).

Lake Qinghai locates at the northeastern Qinghai-Tibet plateau and is hydrologically closed [6] (Fig. 1). The lake surface area is about 4400 km^2 ^and the catchment area is about 29,660 km^2^. Bedrocks of the catchment consist of sedimentary rocks, metamorphic rocks, and a small fraction of igneous rocks. Quaternary loess is distributed around the lake, and seasonal frozen earth is widely distributed in the valleys of the catchment [7].

Annual mean temperature is -0.44°C during 1960~2000 (based on the meteorological records from the Gangcha station which is about 10 km north of Lake Qinghai). The warm seasons are during May to September, with the warmest months of July and August (the averaged temperature is higher than 10°C). The lake is frozen during December to March. Mean annual precipitation is about 300-400 mm, and more than 80% of the precipitation falls between June and September. Evaporation of the lake water is dominated by temperature variations [6]. Water lost by evaporation is much bigger than the amount of inflowing surface water and precipitation; this inequilibration is more striking when temperature is higher. Therefore, the salinity of the lake water is indirectly dominated by temperature variations [8].

Our most recent work showed that the total dissolved solid (TDS) of river waters during the wet period are much higher than during the dry period, suggesting that the chemical weathering is much stronger during wet period than during dry period [9]. Melt of the frozen earth during warm seasons has been suggested to be responsible for this difference. In warm season, the surface frozen earth is melt due to the increase of surface soil temperature; meanwhile both the precipitation and the surface flow increase. As a result, much more terrigenous detritus and dissolved solutes will be brought into the lake, resulting in much higher TDS of the river water during wet seasons than during dry seasons [9].

### Sampling and experiments

Surface cores (about 20~40 cm long) were collected undisturbedly using a self-designed gravity corer, July 2007 (Fig. 1). For each site, we choose a core and cut it into slices every 1 cm in field. We also prepared a mixed surface sample for each site by mixing the whole surface core (referred to as "Mixed-core" subsequently). All of the samples were freeze-dried and then grounded to pass 200-mesh sieves. Sedimentation rates of each site were obtained by ^137^Cs-dating method (refer to [7] for details). Elements contents of the mixed-cores were measured to investigate the spatial distribution patterns of elements in surface sediments. We also measured elements contents of the 1963-section (section containing the 1963 ^137^Cs-peak) of each core to check whether the spatial patterns are also the case for sediments deposited at the same period. Elements contents of some selected 1 cm-sampled cores (shown as black filled circles in Fig. 1) were investigated to get the temporal distribution patterns. River sediments and loess in Lake Qinghai catchment were also collected and their element contents were measured. The measurements were carried out on an X-ray fluorescence spectrometer (XRF, Axios advanced, PW4400). Magnetic susceptibility of samples from each mixed-core was measured (Bartington Meter, Model MS2) to indicate the mean contents of magnetic minerals in the surface sediments.

## Results

### Spatial distribution of Rb/Sr ratio and major elements

**Figure 2:**
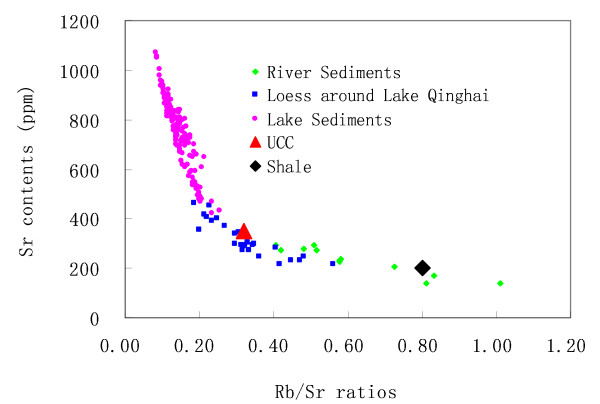
**Plots of Rb/Sr ratios vs. Sr contents**. The mean Rb/Sr ratio of the surface sediment in Lake Qinghai is less than 0.15. The mean Rb/Sr ratios of loess in Lake Qinghai catchment and of the river sediments are 0.33 and 0.62, respectively, which are similar to the Rb/Sr ratio of upper continental crust (UCC, 0.32) and that of the shale (0.80). Sr contents and the Rb/Sr ratios of upper continental crust (UCC) and shale are from reference [4].

The Sr contents of the sediments of Lake Qinghai are much higher than those of the loess around. It is also higher than Sr contents of loess in other regions in China [e.g. [10,11]], and higher than Sr contents of the upper continental crust (UCC) and that of the terrigenous shale [4]. As shown in Fig. 2, the Rb/Sr ratios of the bulk lake sediments, river sediments, and loess are significantly different. The average Rb/Sr ratio of the river deposits in Lake Qinghai catchment is 0.62, which is higher than the average Rb/Sr ratios of loess (0.33) and that of the lake sediments (< 0.15). This is possibly because the river sediments are leached more during the transportation processes due to the larger water-particle interface.

Elements contents of the surface sediments of the mixed-cores and of the1963-section of each core are presented in Table 1. The elements can be generally classified into two groups: Group I(SiO_2_, Al_2_O_3_, Fe_2_O_3_, K_2_O, Ti, etc.) and Group II(CaO, Na_2_O, Sr). Elements in Group I are mainly of terrigenous provenance, and they are inversely correlated with those in Group II; the latter are mainly chemically and/or biogenically deposited (Table 2). Ca is highly correlated with Sr, consistent with their similar geochemical behavior. The Rb/Sr ratios are positively correlated with elements in Group I, while negatively correlated with those in Group II(Table 2).

**Table 1:** Major element contents of surface sediments in Lake Qinghai

		*Surface sediments in Lake Qinghai*							
		*QH0710*	*STS*	*QH0708*	*QH0706*	*QH0707*	*QH0711*	*QH0709*	*#1*
SiO_2_(%)	Mixed-core	48.8	50.8	43.0	39.9	38.6	38.0	37.4	36.7
	1963-sect.	48.2	48.6	41.4	39.9	40.8	38.6	35.6	36.8
***Al_2_O_3_(%)***	***Mixed-core***	***11.4***	***9.9***	***9.6***	***10.1***	***9.5***	***9.7***	***9.0***	***9.5***
	***1963-sect*.**	***10.6***	***10.3***	***9.3***	***9.3***	***9.6***	***8.9***	***8.5***	***9.4***
Fe_2_O_3_(%)	Mixed-core	4.6	4.2	4.1	4.1	3.9	3.9	3.7	3.8
	1963-sect.	4.2	4.3	4.1	4.0	4.1	3.8	3.5	3.6
***MgO(%)***	***Mixed-core***	***4.0***	***4.5***	***4.1***	***3.8***	***3.5***	***3.6***	***3.7***	***3.4***
	***1963-sect*.**	***3.8***	***4.1***	***4.2***	***4.4***	***4.1***	***4.2***	***3.9***	***3.6***
CaO(%)	Mixed-core	12.3	10.7	16.3	18.6	19.2	20.5	21.3	21.7
	1963-sect.	12.9	12.6	17.2	17.8	16.8	19.9	22.6	21.8
***Na_2_O(%)***	***Mixed-core***	***2.1***	***2.1***	***2.5***	***2.4***	***2.3***	***2.7***	***2.3***	***2.5***
	***1963-sect*.**	***2.3***	***2.4***	***2.6***	***2.8***	***3.1***	***2.7***	***2.6***	***2.7***
K_2_O(%)	Mixed-core	2.5	2.1	2.1	2.2	2.1	2.1	2.0	2.1
	1963-sect.	2.3	2.2	2.0	2.1	2.1	2.0	1.9	2.1
***Ti(ppm)***	***Mixed-core***	***3498.1***	***3472.5***	***3145.8***	***2960.5***	***2741.8***	***2814.4***	***2755.1***	***2720.8***
	***1963-sect*.**	***3342.6***	***3455.2***	***3062.7***	***2906.8***	***2975.0***	***2797.7***	***2544.0***	***2677.4***
Rb(ppm)	Mixed-core	102.4	98.0	104.3	112.5	113.1	100.5	95.7	101.3
	1963-sect.	97.9	98.0	103.4	129.9	117.4	116.2	94.8	92.5
***Sr(ppm)***	***Mixed-core***	***354.7***	***400.0***	***618.0***	***726.1***	***805.3***	***772.1***	***832.4***	***869.0***
	***1963-sect*.**	***385.3***	***449.5***	***662.6***	***700.4***	***648.6***	***782.8***	***920.9***	***824.1***

Mixed-core represents the mixture of the surface core. 1963-sect. represents the section of each core containing the ^137^Cs peak.

**Table 2:** Correlation coefficients between major elements of surface sediments in Lake Qinghai

	Al_2_O_3_ (%)	Fe_2_O_3_ (%)	K_2_O (%)	Ti (ppm)	Rb (ppm)	MgO (%)	Na_2_O (%)	Sr (ppm)	CaO (%)	Rb/Sr
SiO_2_	**0.655**	**0.841**	**0.553**	**0.983**	*-0.198*	**0.891**	*-0.702*	*-0.984*	*-0.995*	**0.953**
Al_2_O_3_		**0.944**	**0.989**	**0.732**	**0.124**	**0.319**	*-0.460*	*-0.760*	*-0.651*	**0.838**
Fe_2_O_3_			**0.897**	**0.901**	**0.054**	**0.589**	*-0.560*	*-0.910*	*-0.841*	**0.941**
K_2_O				**0.635**	**0.226**	**0.202**	*-0.421*	*-0.669*	*-0.555*	**0.765**
Ti					*-0.194*	**0.866**	*-0.651*	*-0.993*	*-0.975*	**0.962**
Rb						*-0.253*	**0.020**	**0.181**	**0.113**	*-0.106*
MgO							*-0.580*	*-0.836*	*-0.890*	**0.732**
Na_2_O								**0.660**	**0.700**	*-0.724*
Sr									**0.977**	*-0.980*
CaO										*-0.947*

Bold and italic fonts show positive and negative correlations respectively. The underlined correlation coefficients are higher than 0.6.

**Figure 3:**
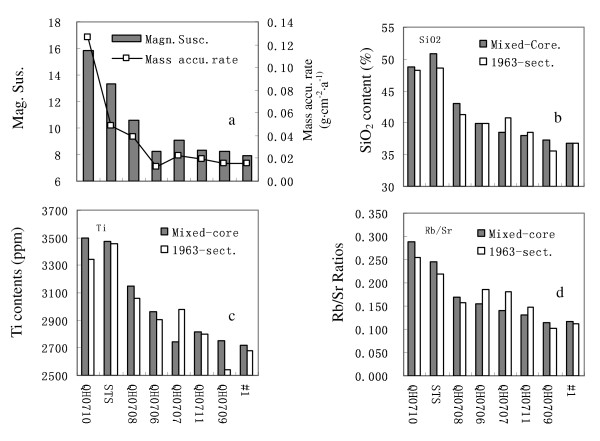
**Spatial patterns of mass accumulation rates and magnetic susceptibility (a), SiO_2 _(b) and Ti (c) contents, and Rb/Sr ratios (d) of the surface sediments in Lake Qinghai**. The horizontal coordinates are arranged according to both mass accumulation rate and the distance from sampling sites to the estuaries and/or lake shoreline. The magnetic susceptibility is highly positively correlated with the corresponding Fe contents (r = 0.9; figure not shown). Elements of the mixed-cores and those of the 1963-sect for each site have similar spatial patterns.

As shown in Fig. 3, the spatial patterns of mass accumulation rate, magnetic susceptibility, major elements, and Rb/Sr ratios are similar. Our most recent work revealed that SiO_2_, Ti, Fe, Al_2_O_3 _are the main components of lake sediments [7]. These elements have higher contents near bank/estuary areas, while lower at the central region. Therefore, mass accumulation rates of surface sediments are mainly dominated by accumulation of terrigenous detritus (Fig. 3; refer to [7] for more details). On the contrary, the chemically/biogenically deposited components have lower contents near bank/estuary areas but higher at central lake. These suggest that the spatial pattern of Rb/Sr ratios are dominated by input of terrigenous detritus.

### Temporal patterns of Rb/Sr ratios of the surface sediments

**Figure 4:**
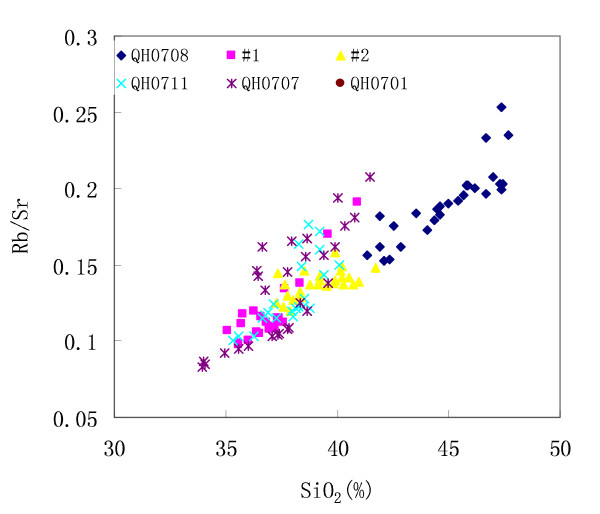
**Temporal relationship between Rb/Sr ratios and terrigenous fraction (exemplified by SiO2 contents here) for core #1, #2, QH0701, QH0707, QH0708, and QH0711**.

Fig. 4 shows the temporal relationship between Rb/Sr and SiO_2 _contents. For each individual core, the Rb/Sr ratios are positively linearly correlated with SiO_2 _contents. This positively linear relationship also exist between Rb/Sr ratios and other elements in Group|(i.e. Al_2_O_3_, Fe_2_O_3_, K_2_O, Ti, etc. Figures not shown), suggesting that high fraction of terrigenous detritus correlates with high Rb/Sr ratios of the bulk sediments. This is consistent with the spatial relationship between Rb/Sr ratios and SiO_2 _and Ti contents (Fig. 3).

**Figure 5:**
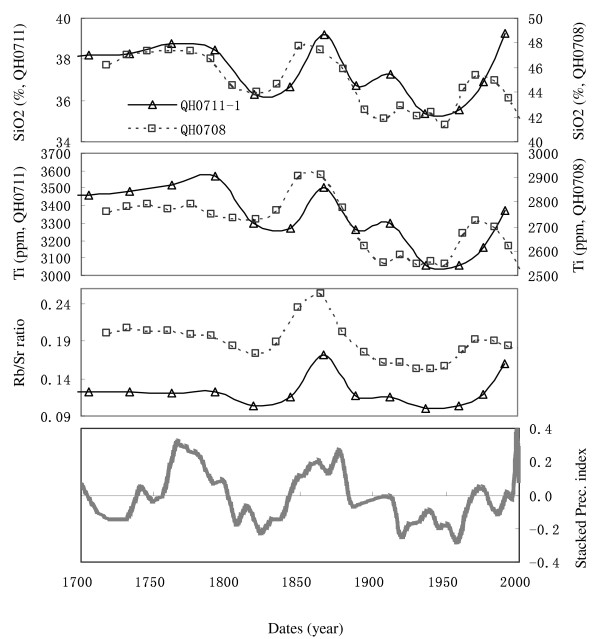
**Comparisons between SiO_2 _and Ti contents, Rb/Sr ratios of core QH0708 (thin line, triangle) and core QH0711 (dashed line, square), and the stacked precipitation index (thick curve)**. The stacked precipitation index is the average of the normalized C/N_org_, the detrended total organic carbon (TOC_detrended_), and -δ^13^C_org _(see [8,12] for details). Note that the element contents of Core QH0708 are systematically higher than those of QH0711, consistent with the corresponding spatial patterns (see Fig.3).

Our previous work showed that variations in δ^13^C of the organic matter (δ^13^C_org_), C/N atomic ratio, and the total organic carbon content of the surface sediments in Lake Qinghai are dominated by local precipitation (see [8,12] for details). As shown in Fig. 5, the trends of Rb/Sr ratios of core QH0708 and core QH0711 are generally similar with those of the precipitation indicators. On decadal scales, higher precipitation correlates with higher Rb/Sr ratios of the sediments. The SiO_2 _and Ti contents are also consistent with the precipitation trend, suggesting that the surface sediments contain higher proportion of terrigenous detritus when the local precipitation is higher. However, the other cores do not show such relationship, which are possibly due to the dilution of chemical/biogenic deposits (see next).

## Discussions

### Rb/Sr ratios in sediments and physical weathering

The Sr-bearing minerals in sediments are from two parts, one from the terrigenous detritus and another from the chemical/biogenic deposits. If the Rb/Sr ratios of the sediments are dominated by physical weathering, then stronger physical weathering should correlate to higher fraction of terrigenous detritus and therefore to higher Rb/Sr ratios of lake sediment. On the contrary, if the Rb/Sr ratios are mainly determined by chemical/biogenic deposits, then stronger chemical weathering may correlate to higher Sr contents in lake sediment, and therefore to lower Rb/Sr ratios. This means that the physical weathering and chemical weathering should be opposite and compete in controlling the Rb/Sr signals of lake sediments. As shown in Fig. 3 and Fig. 4, the spatial and temporal patterns of Rb/Sr ratios are positively correlated with those of SiO_2 _and Ti, suggesting that the Rb/Sr ratios of lake sediments may be dominated by proportions of terrigenous detritus, and therefore by physical weathering within the catchment.

Why does the Rb/Sr ratio in sediments of Lake Qinghai mainly reflect the intensity of physical weathering on decadal scales? This should be ascribed to the physical/chemical properties of the catchment and the local environmental processes. One of the important factors influencing the Rb/Sr ratios of Lake Qinghai may be the widely distributed seasonal frozen earth in the catchment. As shown by previous work, temperature and precipitation are generally synchronous at Lake Qinghai on decadal scale [8,12]. When temperature is higher, more of the surface frozen earth melts; meanwhile the increasing precipitation at the same period will enhance the transportation of terrigenous detritus. As a result, more terrigenous detritus would come into the lake when precipitation is increased, resulting in higher Rb/Sr ratios of the bulk lake sediments.

### Relationship between Rb/Sr ratios and weathering intensity on different timescales

On short term timescales (e.g. decadal scale), when physical weathering increases, the chemical weathering intensity in Lake Qinghai catchment is expected to increase because of the increased temperature and precipitation and because of the stronger hydro-rock reaction since more terrigenous particles are involved. The dissolved Sr concentration of river waters in Lake Qinghai catchment is 0.11 mg/L during the dry season, and increases to 0.22 mg/L during July to September, consistent with the higher TDS of river waters in wet seasons than in dry seasons [9]. However, the chemically deposited Sr-bearing mineral is expected to be diluted by the increased proportion of terrigenous detritus when physical loads increases on short term timescales. This implies, during the wet-warm intervals, even though the intensity of chemical weathering increase in Lake Qinghai catchment, the contents of chemically/biogenically deposited Sr-bearing minerals in lake sediments would decrease rather than increase. The inversely correlated spatial patterns between the flux and contents of CaCO_3_, which is mainly chemically/biogenically deposited, support such an inference (see [7] for details). Therefore, the increased chemical weathering would also correlate to higher Rb/Sr ratios on short term timescales, e.g. decadal scale.

However, on long term timescales such as millennium scale, chemical weathering may be more important to regulate the long term trend of Rb/Sr ratios in lake sediment. The water chemistry indicates that the concentration of Ca^2+ ^of the lake water is lower than those of the river water and ground water, which is because of the fast deposition of Ca as CaCO_3 _due to the high pH and high salinity of the lake water [9]. This means the long term variation of CaCO_3 _flux, as well as that of Sr-bearing minerals, is determined by the material influx, which is closely related to both temperature and precipitation. Meanwhile, the deposition rate of autogenic/biogenic carbonates at Lake Qinghai is influenced by the temperature-dominated water salinity (as mentioned in the background section); the long term trends of Sr contents and the Rb/Sr ratios in the lake sediments should contain combined climatic signals of both precipitation and temperature. The highly coherence between Rb/Sr ratios and precipitation-or temperature-indices on millennial timescales supports our inference (Lake Qinghai deep drilling program, unpublished data). Therefore, considering the relationship between precipitation and Rb/Sr ratios, we can get a relationship of "higher precipitation ~ lower Rb/Sr ratios" on long term timescale, which is just opposite with the relationship of "higher precipitation~ higher Rb/Sr ratios" on decadal scale (as shown in Fig. 5). As a result, different timescale must be considered when evaluating the geochemical significance of the Rb/Sr ratios in lake sediments.

### How well can the Rb/Sr ratio reflect the weathering intensity?

Since the physical weathering and chemical weathering in Lake Qinghai catchments may have opposite influence on Rb/Sr ratios of the bulk sediments, and they are competing in dominating the Rb/Sr ratios of lake sediments on different timescales, the Rb/Sr ratios of sediments in Lake Qinghai may not always faithfully reflect the intensity of weathering in the catchment and the related climatic changes. When precipitation increases, both the flux of terrigenous detritus and the concentration of Sr in river waters in Lake Qinghai catchment increase. The Sr contents in sediments near the estuary/bank areas will be diluted by other major minerals as mentioned above. However, the Sr contents in sediments in the central lake may not be affected so strongly. This means that the Sr contents may exhibit different patterns between different regions in the lake. As a result, both the Sr content and the Rb/Sr ratios of sediments near the estuary/bank areas may be different with those of sediments at the central lake. This is why that the Rb/Sr ratios of only two cores show similar trends with the precipitation indices while others not (Fig. 5).

Other factors such as the grain size of the sediments, the lake level, etc., can also influence the Rb/Sr ratios of lake sediments. For instance, the increase and decrease of lake level will change the distance from a given site to the estuaries and lake shores. Therefore, both the flux and grainsize of terrigenous detritus are expected to vary with the increase and/or decrease of the lake level for a certain site, and the Rb/Sr ratios of sediments collected there should vary consequently. As a result, the Rb/Sr ratios of lake sediments should be cautiously used as an indicator of weathering intensity in the catchments.

## Summary

We investigated the spatial and temporal patterns of major elements in surface sediments of Lake Qinghai. The spatial pattern of Rb/Sr ratios of the bulk sediments correlates well with the spatial pattern of mass accumulation rate, and those of the SiO_2 _and Ti contents. The decadal variations of Rb/Sr ratios are also positively correlated with those of SiO_2 _and Ti contents for each individual core, suggesting that Rb/Sr ratios of the surface lake sediments are dominated by the proportion of terrigenous detritus, which is closely related to both precipitation and temperature variations.

However, factors influencing Rb/Sr ratios of lake sediments are very complex. The physical/chemical properties of the bedrocks in catchment, the local climates, and even the sampling site within a lake would affect the environmental significance of Rb/Sr ratios. Physical weathering and chemical weathering generally have opposite influence on Rb/Sr ratios of the bulk sediments, and they may compete in dominating the Rb/Sr ratios of lake sediments on different spatial and temporal scales. As a result, particular investigations of the spatial and temporal (especially short term timescale) patterns of the Rb/Sr ratio are necessary before using the Rb/Sr ratios of the lake sediments to study the weathering intensity in the catchment.

## Competing interests

The authors declare that they have no competing interests.

## Authors' contributions

**HX **carried out field work, experiments and drafted the manuscript. **BL **carried out the XRF measurements of lake sediments. **FW **provided the XRF data of river sediments and loess. All authors read and approved the final manuscript.
